# Charge Distribution Dependent Spectral Analysis of the Oxidized Diferrocenyl-Oligothienylene-Vinylene Molecular Wires

**DOI:** 10.1038/srep35726

**Published:** 2016-10-19

**Authors:** Lixin Xia, Jing Wang, Caiqing Ma, Shiwei Wu, Peng Song

**Affiliations:** 1 Department of Chemistry, Liaoning University, Shenyang 110036, P. R. China; 2 Department of Physics, Liaoning University, Shenyang 110036, P. R. China

## Abstract

The vibrational spectra have been investigated for revealing the comprehensive structure of diferrocenyl-oligothienylene-vinylene complex, stimulated by the excellent experimental reports [group of Casado J. Am. Chem. Soc. 2012, 134, 12, 5675]. The IR and Raman spectra were simulated. It is found that the change of charge distribution and bond length are associated with the variation in the frequencies of specific vibration in infrared spectra for the neutral and radical oxidation states. The theoretical simulation of charge difference density indicate that charge transfer mechanism for neutral and dication states are significant different. The results can offer hints for the rational design of novel and interesting oligomer semiconductor.

The research of organic π−conjugated molecules are emerged spanning chemistry, physics and material science, due to its main application in hole transporter (p-type materials). Various well-design molecules and remarkable researches have been done in this field^1^,^2^. Oligothiophenes and its derivatives are involed in an interesting class of the semiconductor material. As reported, sixithiophene can be used as the active materials^3^. Its backbone can easily be modified with aromatic group and olefin for obtaining more chemical stability and rigid molecular structure^4^,^5^,^6^. This result in oligothiophenes and polymer thereof are the prime design and synthetic targets in the fields. For example, to improve the electronic transition ability, the oligothiophenes backbone with two different capping-ends has been synthesized and the metals were introduced in. On the basic research side, π−conjugated materials with the interplay between their π-electronic structure and their geometric structure have attracted many attentions. The structure and electrons states have been investigated to get hints for designing new types of molecule^7^,^8^.

In 2012, group of Casado reported the excellent work on their well-designed molecule, Fc-nTV-Fc. They imported ferrocene (FC) group as two capping-end and oligothiophene-vinyl (TV) wire as a mediating molecular bridge^9^. Moreover, the Raman spectra were employed to characterize the change of thienyl/vinyl, the role of ferrocenyl, and further the charge transition were explored. Their results reveal that the molecule has a turning point of the electron delocalized and localized distribution when n = 6. The distribution was influenced by solvent, temperature and oxidation. The conjugated structure of molecule equalized the charge distribution between single bonds and double bonds of the oligothiophene and vinyl wire. Furthermore, another well-designed molecule oligofurans and oligothiophenes with ferrocene as capping-end was obtained, and stronger charge delocalization in ferrocene-capped oligofurans are found^10^. Recently, J. Casado and coworkers investigated the properties of carbon-bridged oligo (para-phenylenevinylene)s (COPVn), which exerts extensive charge delocalization^11^.

It should be noted that, in order to understand the relationship between the molecular structure with its optical and electronic properties, it is necessary to study the whole vibrational spectra^12^. Theoretically, a nonlinear molecule has independent 3N−6 modes of vibration. IR spectra can present the vibrational motions accompanied the change of dipole moment, while Raman spectra can provide other motions related to the change of polarizability. So combining the IR and Raman spectra one can obtain the comprehensive information of the complex structure, which are referred to as finger-print spectra.

In this paper, we theoretically characterize the structural, vibrational spectra and electronic properties of Fc-nTV-Fc in neutral and different oxidation states. Firstly, the optimized structure in neutral and oxidation states was studied by density functional theory (DFT) method. Secondly, the optical spectra were simulated and their vibrational modes were analyzed. Furthermore, the charge transfer, calculated by Multiwfn program^13^, was employed in investigating the electronic distribution states. Here, we pay more attention on the IR spectra and their vibration modes when n = 6, due to its obvious Raman spectra results in the previous study^9^.

## Results and Discussions

### The vibrational spectra of Fc-nTV-Fc with different n

**Figure 1:**
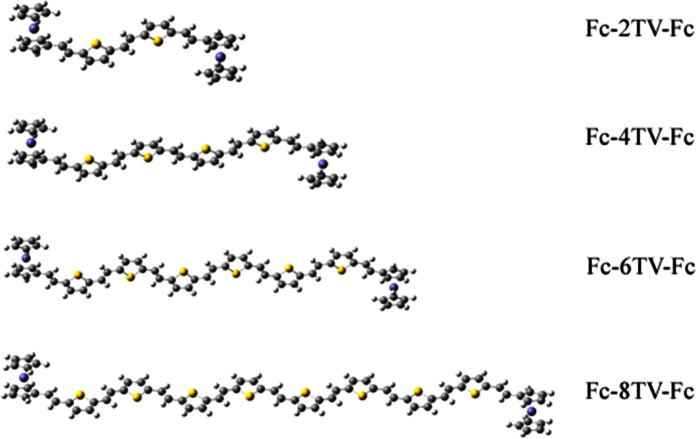
The optimized geometries of neutral molecules. The gray balls represent C atoms. The yellow and purple ones stand for the S atoms and Fe atoms, respectively.

**Figure 2:**
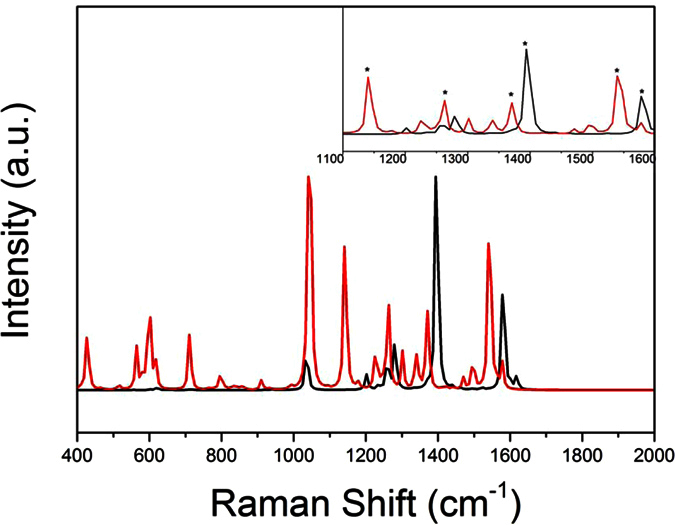
The normalized calculated Raman spectra of Fc-4TV-Fc in neutral (black line) and its cation states (red line). The enlargement curve from 1100–1600 cm^−1^ is shown in the inset.

The optimized geometries of different Fc-nTV-Fc have been obtained by DFT method and shown in Fig. 1. Based on the optimized geometries, the IR and Raman spectra of these systems were simulated and the frequency analysis was also performed, no imaginary frequency was found. Detailed comparison with the experiment indicates that the calculated Raman spectra of Fc-nTV-Fc and its radical cations contrast well with the experimental results^9^. As can be seen from Fig. 2, for Fc-4TV-Fc, there are two obvious peaks in the neutral molecule (black line in Fig. 2), that are 1578 cm^−1^ and 1394 cm^−1^ correspond the experiment measured frequencies 1577 cm^−1^ and 1387cm^−1^, respectively. When Fc-4TV-Fc is in the radical state, the calculated spectra peaked at 1540 cm^−1^, 1370 cm^−1^, 1263 cm^−1^, 1140 cm^−1^ and 1040 cm^−1^, respectively (see the red line in Fig. 2), which associates with the experimental ones at 1548 cm^−1^, 1359 cm^−1^, 1240 cm^−1^ and two weak peaks that fell into the range between 1200 cm^−1^ and 1100 cm^−1^. Detailed analysis reveals that, for the neutral molecule, the spectra located in 1600–1500 cm^−1^ can be assigned to the symmetric vibration of vinylene ν(-C = C-), and in 1500–1300 cm^−1^ is the vibration from thiophenic ν(-C = C-), and in 1300–1200 cm^−1^ assigned to the flexural vibration of vinylene β(H-C). The coincidence between the theoretical results and experimental data indicates that the selected calculate approach is valid.

**Figure 3:**
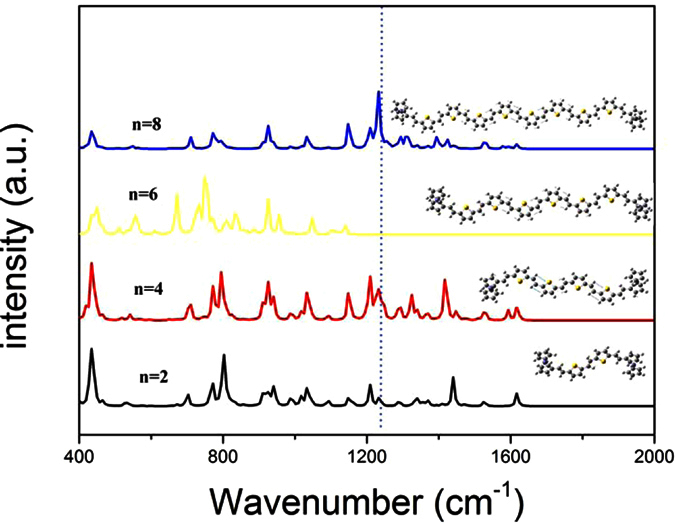
IR spectra of neutral molecules with different n (n = 2, 4, 6, 8). The vibrational motions at the marked wavenumber are listed in the blank.

To supply the experimental information of these complex molecular structures and further investigate the π−conjugated chain dependent comprehensive vibrational spectra, the IR spectra of neutral Fc-nTV-Fc molecules (with n = 2, 4, 6, 8) were calculated. It can be easily found from Fig. 3 that, there is an obvious tendency of increasing the relative intensity around 1230 cm^−1^ with the length of π−conjugated system. This peak is mainly contributed by C = C stretching vibration of the vinyl group. It can be clearly seen from the vibrational mode analysis shown as the inserts above the spectra line in Fig. 3. As a reasonable explanation, for larger TV wire length, much more electron of inner vinyl and thienyl (excluding two vinyl-thienyl units adjacent to ferrocene) delocalizing along the conjugated structures, which make the resonance intensity much higher than that of molecule with lower TV wire length due to its limited range of C = C chain. For comparison, the optimized geometries and calculated vibrational spectra of nTV (n = 2, 4, 6 and 8) are listed in Figs S1 and S2, respectively.

### The vibrational spectra of Fc-6TV-Fc in different oxidation states

**Figure 4:**
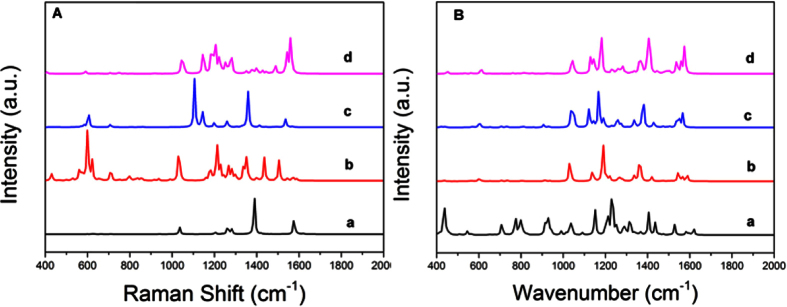
The spectra of Fc-6TV-Fc in neutral and oxidation states. (a–d) present the neutral state and charged states with +1, +2, +3, respectively.

As described above, the interesting property Fc-6TV-Fc reveals that its spectra are significantly affected by the oxidation states and other experimental condition such as temperature and solvent^9^. This performance is highlighted by their ability to transmit the interferrocene coupling over distances near 40 Å, which is an outstanding wirelike feature. Simply discuss here, only the higher oxidation states (+1, +2, +3) were chosen for further investigation. The IR and Raman spectra from 400 cm^−1^ to 2000 cm^−1^ are present in Fig. 4 in contrast to the neutral one. We still focus on the characteristic peak discussed above. The corresponding IR-active motion and frequencies for this peak are listed in Table 1. (For comparison, the vibrational motions of nTV (n = 2, 4, 6 and 8) at the wavenumber around 1200 cm^−1^ and 1230 cm^−1^ are shown in Fig. S3a,b). For higher oxidation, the wavenumber of the highest intensity largely red shifts. Similarly, the molecular structure change of the different oxidation state is expected to be the origin for the frequency shift.

**Figure 5:**
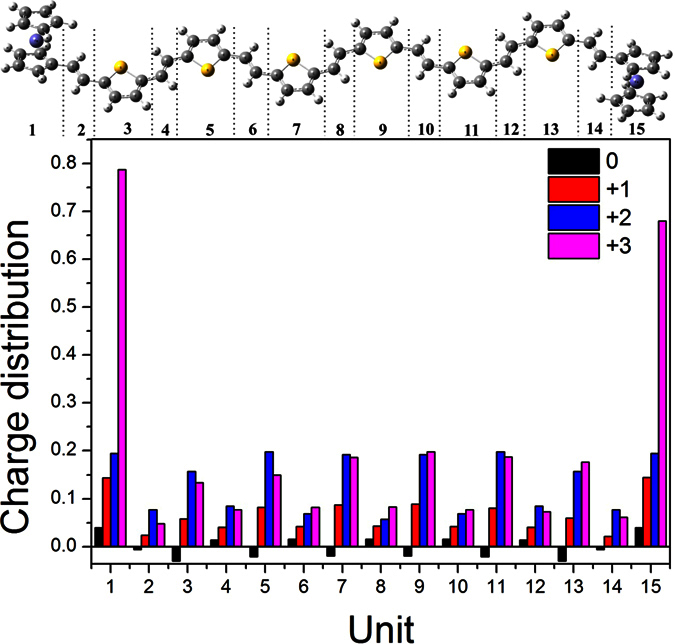
The charge distribution of Fc-6TV-Fc in neutral and oxidation states (Mulliken charge).

Usually, the bond length and charge distribution are important for investigating the change of molecule structure. For Fc-6TV-Fc molecule, it can be partitioned into fifteen parts. The calculated charge distribution (Mulliken charge) are shown in Fig. 5 and the columns in black, red, blue and pink represent the charge distributions of neutral, radical cation, dication and trication, respectively. Obviously, more charges are located on two capping ferrocene units for the neutral one. As the increasing of oxidation states (0, +1, +2), more charge distribute on thienyl instead of ferrocene group. When the state is +2, charge located on single thienyl group is more than single ferrocene group. Moreover, the charges are found to delocalize on the whole units and charge density become weaker on the two ferrocene capping-end. So the radical dication state can be recognized as a turning point. For the +3 one, the distribution emerges a significant difference from the others. In detail, the charges mainly localize on the ferrocene units and these two capping-end do not play an equal role as the charge supporter. Additionally, the charge distribution on the single thienyl unit is even less than that of +2 charged state, though the total charges increasing. So, the fact that charge distribution strongly influenced by the degree of oxidation can be concluded.

**Figure 6:**
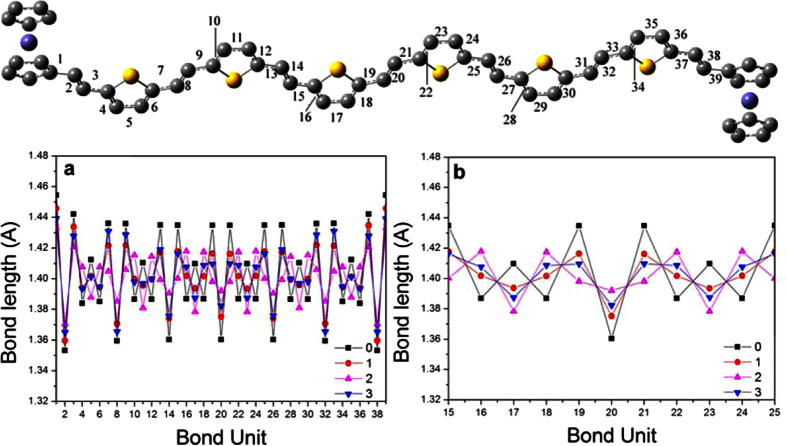
The bond lengths of Fc-6TV-Fc in neutral and oxidation states.

Furthermore, the bond length of the wire units adjusted by oxidation effect was studied. For clear presentation, the C-C bond and C = C bond on the wire was sequentially numbered. As shown in Fig. 6a, the bond length of the neutral molecule (black square) present the typical C-C and C = C characterization. In accordance to the above charge distribution analysis results, the bond length are found to be changed with the state higher oxidized. Also, the most obvious distinction emerges between the radical dication state and the neutral state, and mainly concentrates on the units of 16–18 and 22–24 (C-C and C = C bond of the two intermediate thieyl). These bond lengths display a contrary tendency to the neutral ones (see Fig. 6b). Especially, the bond lengths of 19–21 in dication state are nearly equal, while the neutral state have a large difference between the C-C and C = C. Due to the maximum variation, the radical dication state is also the turning point of bond length. Until now, by combining with the simulated IR spectra result, it can be concluded that the radical dication state shows an interesting property, e.g. the blue shift in frequency and turning point in variation of bond length and charge distribution. This coincides with the experimental conclusion^9^. More charge localization on the center wire results in significant conjugate effect, and further induced the bond length variation and the vibrational frequency shift.

### The photo-induced charge redistribution in the excited states of neutral and dication Fc-6TV-Fc

**Figure 7:**
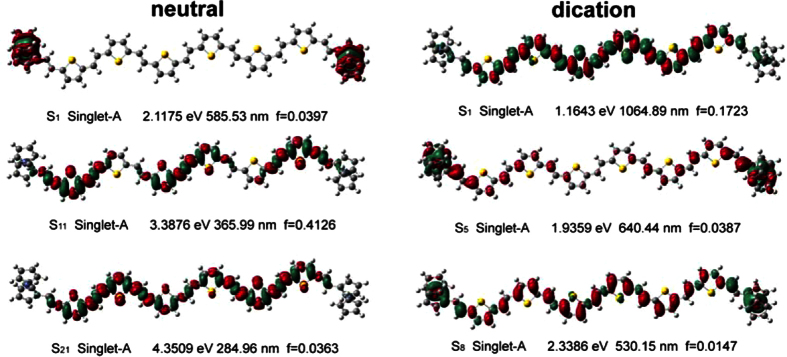
The charge different density of Fc-6TV-Fc in neutral and dication.

The electronic transition of the neutral and charged states was calculated by TDDFT method. 50 excited states were calculated for each molecule. Multiwfn 3.3.1 was employed to obtain the charge difference density (CDD), which can provide a visualized evidence of the electrons and holes redistribution when the molecules are photo excited. We categorized the CDD with higher oscillator strength and the details are listed in Fig. 7 (the green and red represent holes and electrons, respectively). For neutral state of Fc-6TV-Fc, it can be seen in S_1_ state that the electrons and holes are all localized on the capping-end and charge transfer takes place within the redox center. In S_21_ state, electrons and holes distribute on oligomer wire evenly, and only small part of holes localize on the capping-end. While for S_11_ state, the holes and electrons also localize on the conjugated wire, but the distribution were not continuous. Two intervals with week charge or hole transfer are found. When the state is +2 charged (the spin state is same with neutral one, see Table S1), there are alternate distribution of holes and electrons on the oligomer-vinyl wire in S_1_ state (with higher oscillator strength *f* = 0.1723). Most importantly, the electronic transition energy largely red shifts to the infrared region. Detailed analysis reveals that much stronger charge transfer occurs on inner two thienyl-vinyl units. Combined with the vibrational spectra, it can well explain the reason why the dication state of Fc-6TV-Fc shows a biggest IR blue shift at the band of 1168 cm^−1^ from the view point of its vibration motion main contributed by the inner two thienyl-vinyl units. As more charges localize on the wire, the inner two units will show more activity. The S_8_ state displays another distinct type of charge distribution. As shown, the holes mainly localize on the capping-end. This is because the electron transfers from the capping-end to wire for compensating extra charge.

## Conclusions

The structure, vibrational spectra and electronic properties of the oligomer of thienyl-vinyl units with ferrocene capping-end in neutral and different oxidation states were obtained. It is found that the dication state of Fe-6TV-Fe presents the biggest variation in charge distribution, which induces the vibrational frequency blue shift (at 1168 cm^−1^) in IR spectra and bond length change from the neutral state. The first excited state of this dication state with high oscillator strength also shows more activity on the inner two thienyl-vinyl units, which mainly contribute to the vibration at 1168 cm^−1^.

## Method

All the quantum chemical calculations were done withGaussian 09 software. The geometry of the neutral andoxidized molecules at ground state were optimized by DFT method^14^, B3LYP functional^15^,^16^, 6-31G(D) basis set for C H S atoms and LANL2DZ^17^ basis set for Fe atoms. The harmonic vibrational spectra and the excited states were obtained by the same functional and basis set. The final frequencies were scaled by a uniform factor of 0.96. Cam-B3LYP functional was made a comparison in excited states by the long range corrected version of B3LYP, using the Coulomb-attenuating method. This correction makes it more suitable for electron excitation to high orbitals^18^. Visualizations of charge transfer and electron-hole coherence on electronictransitions were done with Multiwfn 3.3.1.

## Additional Information

**How to cite this article**: Xia, L. *et al*. Charge Distribution Dependent Spectral Analysis of the Oxidized Diferrocenyl-Oligothienylene-Vinylene Molecular Wires. *Sci. Rep.* **6**, 35726; doi: 10.1038/srep35726 (2016).

## Supplementary Material

Supplementary Information

## Figures and Tables

**Table 1:**
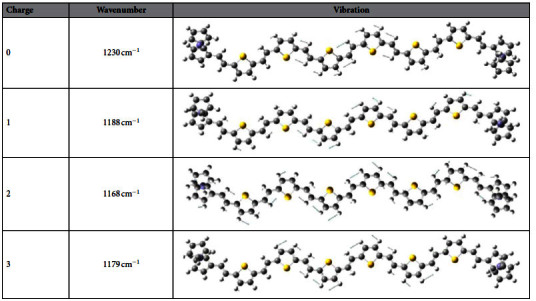
The wavenumber and vibration modes of Fc-6TV-Fc in neutral and higher oxidation.

This vibration is assigned by *β*(C-H).
