# Supplementary material for: Charge Distribution Dependent Spectral Analysis of the Oxidized Diferrocenyl-Oligothienylene-Vinylene Molecular Wires
Source: Sci Rep. 2016 Oct 19;6:35726. doi: 10.1038/srep35726 (PMC5069631; doi:10.1038/srep35726)
Supplement: Supplementary Information [file srep35726-s1.doc]

**Supplementary Information**

**Charge Distribution Dependent Spectral Analysis of the Oxidized Diferrocenyl-Oligothienylene-Vinylene Molecular Wires**

**Contents**

Lixin Xia ^a*^, Jing Wang ^a^, Caiqing Ma ^a^, Shiwei Wu ^a^, Peng Song ^b*^

*^a^Department of Chemistry, Liaoning University, Shenyang 110036, P. R. China*

*^b^Department of Physics, Liaoning University, Shenyang 110036, P. R. China*

Corresponding Authors: lixinxia@lnu.edu.cn (L.X. Xia) and songpeng@lnu.edu.cn (P. Song).

1. Table S1............................................................................................................S1
2. Figure S1..........................................................................................................S2
3. Figure S2...........................................................................................................S3
4. Figure S3...........................................................................................................S4
5. Author contributions.........................................................................................S5

Table S1 The spin state of Fc-6TV-Fc

| Charged state | 0 | +1 | +2 | +3 |
| --- | --- | --- | --- | --- |
| Spin state | 1 | 2 | 1 | 2 |

**
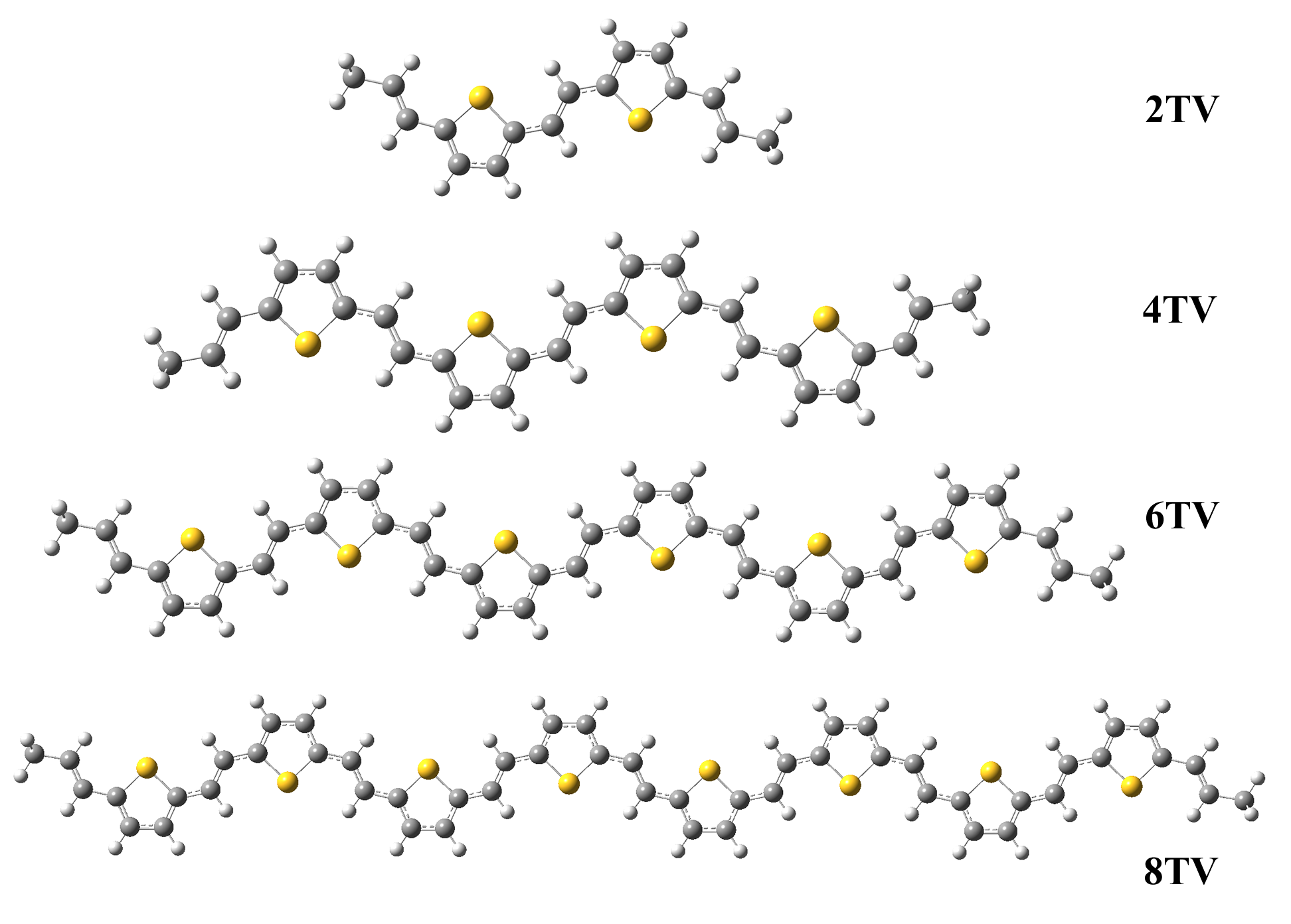
**

**Figure S1.** The geometry of oligomer with different thienyl-vinyl unit (n = 2, 4, 6 and 8)

**
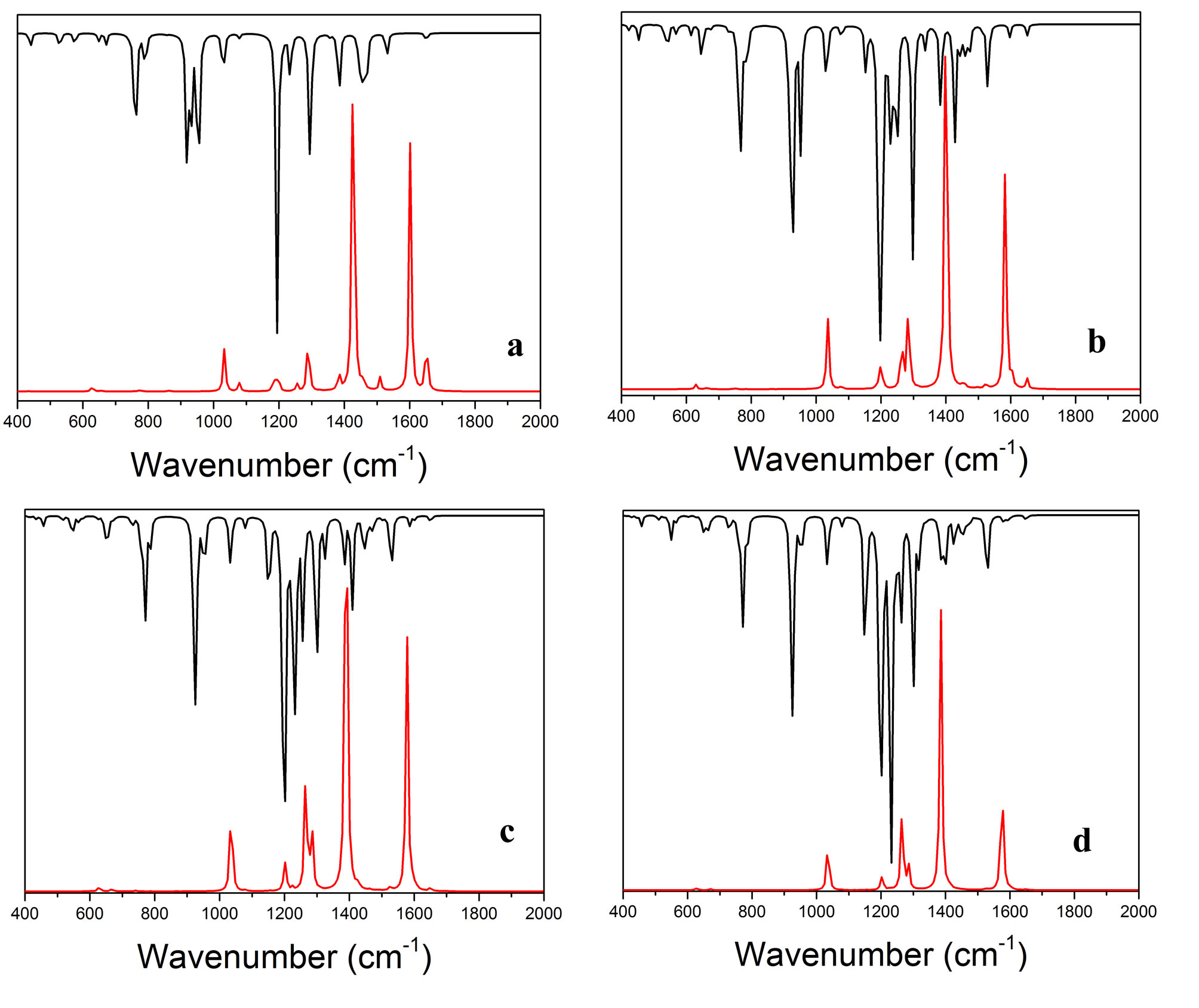
**

**Figure S2.** Calculated IR and Raman spectroscopy of nTV model. The black line on the top is the IR spectra and the red one represents the Raman spectra.


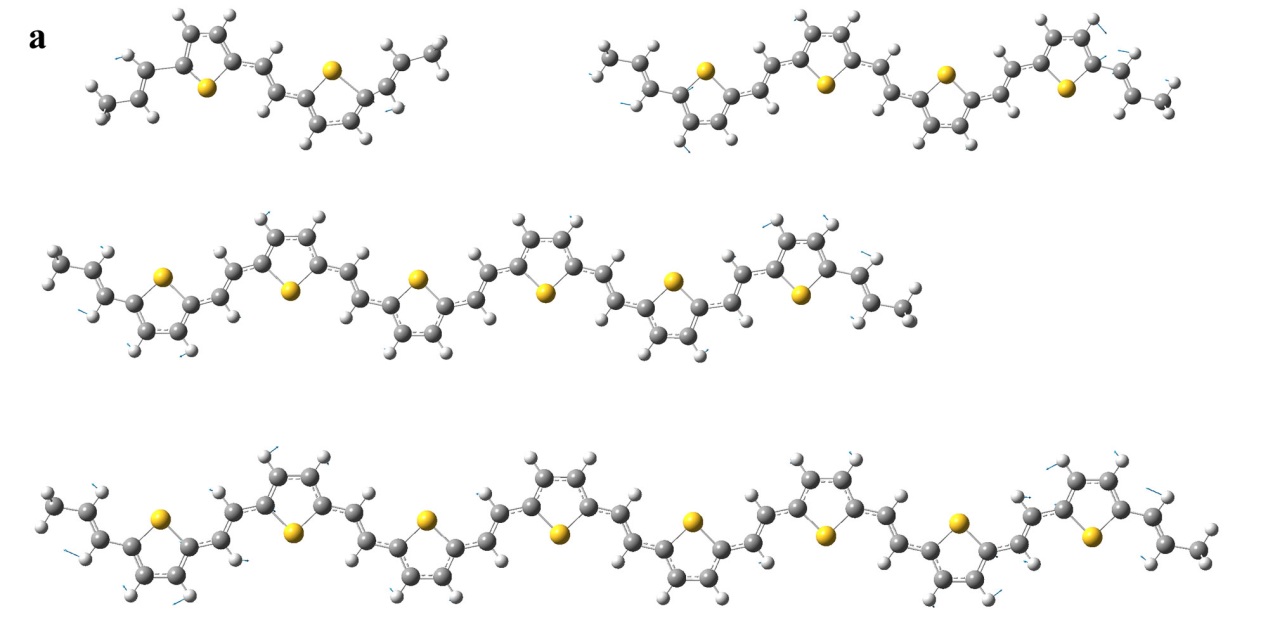


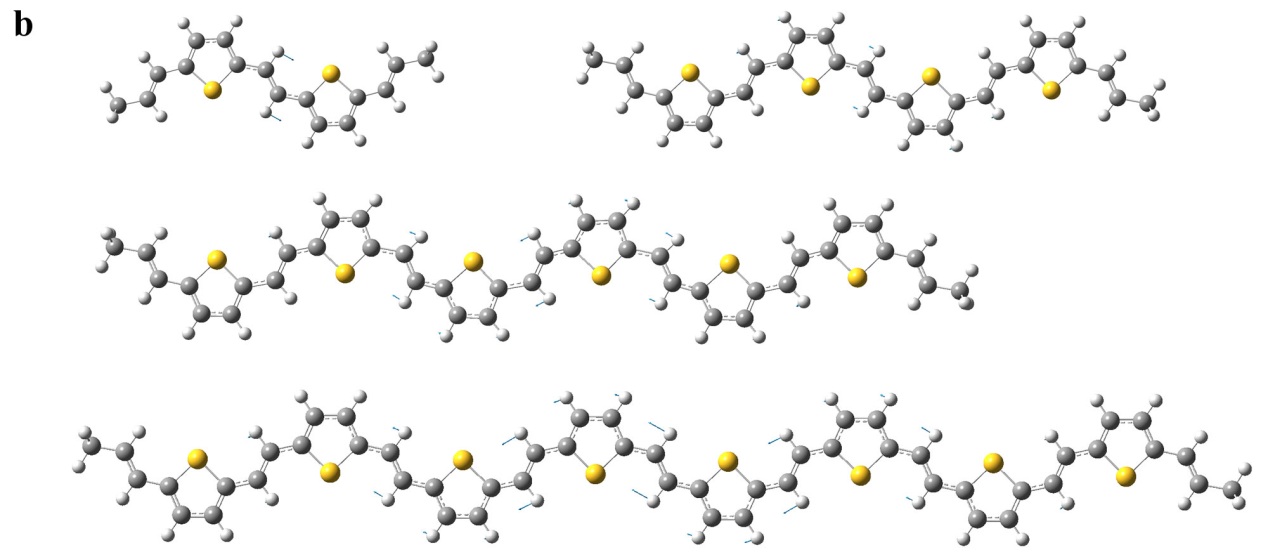


**Figure S3.** The vibrational motions of nTV (n=2, 4, 6 and 8) at the wavenumber around 1200 cm^-1^ (a) and 1230 cm^-1^ (b) in Fig. S2.

**Author contributions**

P. Song and L. X. Xia. supervised the project, J. Wang, C. Q. Ma and S. W. Wu performed the calculations, J. Wang, P. Song and L. X.Xia analysed the data and wrote the paper.
